# Recurrent inhibition of mitochondrial complex III induces chronic pulmonary vasoconstriction and glycolytic switch in the rat lung

**DOI:** 10.1186/s12931-018-0776-1

**Published:** 2018-04-23

**Authors:** Olga Rafikova, Anup Srivastava, Ankit A. Desai, Ruslan Rafikov, Stevan P. Tofovic

**Affiliations:** 1 Division of Endocrinology, Department of Medicine, The University of Arizona, Tucson, AZ 85724 USA; 2 Department of Medicine, The University of Arizona, Tucson, AZ USA; 3 Vascular Medicine Institute, Division of Pulmonary, Allergy and Critical Care Medicine, Department of Medicine, University of Pittsburgh, Pittsburgh, PA USA

**Keywords:** Pulmonary hypertension, Glycolytic switch, Mitochondrial complexes, Protein nitration, Metabolomics

## Abstract

**Background:**

Pulmonary arterial hypertension (PAH) is a fatal disease; however, the mechanisms directly involved in triggering and the progression of PAH are not clear. Based on previous studies that demonstrated a possible role of mitochondrial dysfunction in the pathogenesis of PAH, we investigated the effects of chronic inhibition of mitochondrial function in vivo in healthy rodents.

**Methods:**

Right ventricle systolic pressure (RVSP) was measured in female rats at baseline and up to 24 days after inhibition of mitochondrial respiratory Complex III, induced by Antimycin A (AA, 0.35 mg/kg, given three times starting at baseline and then days 3 and 6 as a bolus injection into the right atrial chamber).

**Results:**

Rodents exposed to AA demonstrated sustained increases in RVSP from days 6 through 24. AA-exposed rodents also possessed a progressive increase in RV end-diastolic pressure but not RV hypertrophy, which may be attributed to either early stages of PAH development or to reduced RV contractility due to inhibition of myocardial respiration. Protein nitration levels in plasma were positively correlated with PAH development in AA-treated rats. This finding was strongly supported by results obtained from PAH humans where plasma protein nitration levels were correlated with markers of PAH severity in female but not male PAH patients. Based on previously reported associations between increased nitric oxide production levels with female gender, we speculate that in females with PAH mitochondrial dysfunction may represent a more deleterious form, in part, due to an increased nitrosative stress development. Indeed, the histological analysis of AA treated rats revealed a strong perivascular edema, a marker of pulmonary endothelial damage. Finally, AA treatment was accompanied by a severe metabolic shift toward glycolysis, a hallmark of PAH pathology.

**Conclusions:**

Chronic mitochondrial dysfunction induces the combination of vascular damage and metabolic reprogramming that may be responsible for PAH development. This mechanism may be especially important in females, perhaps due to an increased NO production and nitrosative stress development.

## Background

Pulmonary arterial hypertension is tightly associated with mitochondrial dysfunction [1, 2]. There are several different mechanisms of mitochondrial dysfunction that have been proposed to be involved in PAH pathogenesis, such as electron transport chain (ETC) dysfunction [3, 4], insufficient mitochondrial biogenesis [5, 6], altered mitochondrial membrane potential [7], increased mitochondrial reactive oxygen species (mROS) production [8, 9], mitochondrial DNA damage [10], and insufficient mitochondrial repair via fission/fusion or mitophagy [11–13]. Most of those mechanisms are also interconnected in many ways. For example, an increased mitochondrial ROS and DNA damage could initiate the processes of fission/fusion, apoptosis or impaired biogenesis. The current work focuses specifically on ETC dysfunction based on its important roles in both ATP production by mitochondrial respiration and as a primary contributor to an increase in mitochondrial ROS. The resulted oxidative stress is known to be strongly incorporated into PAH pathogenesis due to its ability to induce DNA and protein damage. Finally, ETC dysfunction mediates an increase in the mitochondrial potential that has been previously described to mediate a glycolytic shift [14].

There are several clinical cases reporting that mitochondrial respiratory chain disease is accompanied by a manifestation of primary pulmonary hypertension (PPH) and severe pulmonary hypertension [15, 16]. The assessment of the respiratory complexes activity indicated a decreased Complex I and III activity in the tissues of these patients (liver tissue upon liver transplantation, skeletal muscle and skin fibroblasts obtained through biopsy). Thus, the inhibition of Complex III activity is a physiologically relevant stimulus to investigate whether ETC inhibition can induce pulmonary hypertension in a pre-clinical model. Mitochondrial respiratory complexes utilize iron-sulfur clusters to transfer electrons from donors to acceptors. It was also reported that the mutations in the iron-sulfur chaperon protein NFU1 have a strong association with pulmonary hypertension development [17]. All seven patients with NFU1 mutation developed fatal encephalopathy and pulmonary hypertension. Assessment of mitochondria respiratory complexes showed a decrease in Complexes I, II and III activities in patients with the NFU1 mutation, thus, pointing on respiratory chain failure as an important contributor in the development of pulmonary hypertension.

ETC can be affected by inhibition of any of its four known complexes. However, inhibition of Complex III was found to induce maximal ROS production and therefore maximal damage to mitochondria [18]. Electrons flow from Complex I or II to Complex III and then Complex IV generating a gradient of protons in the inner membrane. This gradient is utilized by ATP synthase to produce ATP or by uncoupling proteins to produce heat. Inhibition of Complex III can induce superoxide production in both the intermembrane space and the mitochondrial matrix [19]. Superoxide radical is usually scavenged by a mitochondrial form of superoxide dismutase - MnSOD and transformed into less active hydrogen peroxide (H_2_O_2_) [20]. However, nitric oxide (NO) produced in the vasculature can also scavenge superoxide to form peroxynitrite, a highly potent oxidant, which may inhibit ROS scavengers by induction of protein nitration and nitrosylation, like MnSOD [21], and initiate many pathophysiological pathways [22, 23].

Indeed, lungs of patients with PAH have possessed a ubiquitous nitrotyrosine staining [24, 25], which indicated a high level of peroxynitrite formation in PAH patients. Caveolin-deficient mice that are exhibited chronic pulmonary hypertension and severe right ventricle (RV) hypertrophy [26] had a high level of protein nitration in the lungs due to persistent endothelial nitric oxide synthase (eNOS) activation. Notably, the treatment of Cav1−/− mice with either an eNOS inhibitor or a superoxide scavenger reversed the PAH phenotype. In our recent work, we have reported that nitration mediates an over-activation of Akt with subsequent translocation of eNOS to the mitochondria. This, in turn, reduces the mitochondrial respiration and promotes endothelial dysfunction [27].

The primary female hormone estrogen is known to increase NO production due to an increase in both eNOS expression and activity [28, 29]. The vasodilatory, anti-inflammatory, and anti-thrombotic properties of NO provide the background for an advanced level of cardiovascular protection in females compared to males. At the same time, it could be expected that in the presence of mitochondria dysfunction and increased production of superoxide, the estrogen-mediated elevation of NO bioavailability can turn to be a damaging factor through an enhanced formation of peroxynitrite. Since the nitrosative stress has been linked with PAH development, it is possible that an enhanced protein nitration may explain the higher prevalence of PAH in females compared to males. Therefore, in this study, we utilized female rats to elucidate the consequences of repetitive inhibition of the ETC.

Under normal conditions, cells mainly rely on oxidative phosphorylation due to a highly efficient nature of mitochondria-mediated ATP production. However, the disease conditions associated with mitochondrial damage limits mitochondria’s ability to generate ATP and force the cells to switch to glycolysis [30, 31]. This metabolic shift towards a glycolytic metabolism, known as the Warburg effect, has been described in cancer cells, as well as highly proliferative pulmonary vascular cells [32, 33]. Importantly, in our previous research, we have confirmed that the metabolic transformation occurs even at the early stage of PAH when the physiological manifestation of the disease, such as RV pressure and hypertrophy, are still negligible [34]. Thus, the metabolic alterations significantly precede PAH manifestation and may be considered as a contributing factor for PAH development. The current work confirms that inhibition of the mitochondrial ETC is sufficient to initiate the same metabolic changes as seen in lungs of patients with PAH.

## Methods

### A rat model of pulmonary hypertension

Female Sprague Dawley rats (200–250 g) were obtained from Charles River (Wilmington, MA, USA) and used in this study. Animals were housed at 22 °C, 12-h light cycle, 55% relative humidity, and had free access to standard rodent food and water. All experiments were performed in accordance with the University of Pittsburgh Institutional guidelines for animal welfare and the Animal Care and Use Committee approved experimental protocols. On the day of Antimycin A injection, the animals were randomized to receive either vehicle or AA, while anesthetized (pentobarbital, 45 mg/kg i.p.). The PE-50 catheter and Millar catheter were inserted into the right jugular vein, and PE-50 catheter was advanced into right atrium for i.v. AA injection, while the Millar catheter was used to monitor right ventricular systolic pressure (RVSP) and right ventricular diastolic pressure (RVDP) as described previously [21, 35]. Briefly, a customized pressure transducer catheter (SPR-513; Millar Instruments, Houston, TX, USA) was connected to a Millar MPCU-200 pressure-volume conductance system to monitor RV pressure for 30-min after AA injection.

For acute AA treatment (Group AA 30 min), 30 μl of 0.35 mg/kg AA dissolved in 55% ethanol/45% 0.9% saline was injected as a slow bolus injection; the animal was monitored for 30 min and euthanized. This dose was selected based on preliminary experiments (not shown) and was found to be the lowest effective dose tolerated by rats. A bolus injection given directly into the right atrium was used to maximize the exposure of the pulmonary vasculature to AA and minimize the AA effects on the left ventricle. For chronic studies (Groups 6 days, 12 days and 24 days) after the initial injection of AA, the PE-50 catheter placed into the right jugular vein was fixed to the sternocleidomastoid muscle and advanced subcutaneously to the back of the neck through the incision between the scapulae. The ventral neck incision was closed with the wound clips and the dorsal – with 4–0 silk suture to secure the exterior part of the catheter in place and was used for chronic AA injections on days 3 and 6. The RVSP on days 6, 12 and 24 was measured as described above.

### Measurement of mitochondrial respiration

An XF24 Analyzer (Seahorse Biosciences, North Billerica, MA) was used to measure mitochondrial function in intact cells, in real time. Cells (rat pulmonary artery smooth muscle cells and rat pulmonary artery endothelial cells (Cell biologics)) were seeded into Seahorse Bioscience XF24 cell culture plates at the seeding density of 75,000 cells in 100 μl media and allowed to adhere and grow for 16 h in a 37 °C humidified incubator with 5% CO2. Cells were exposed to Antimycin A (10 μM) through the injection port A of the instrument, and continuous measurements of oxygen consumption rate (OCR) were made in media. The total protein was measured by BCA method, used for normalizing the data. The resultant OCR profile provides information on mitochondrial respiratory responses to AA.

### Chemicals

Antimycin A and ethanol for preparation of the stock solution (10 mg/ml) were purchased in Sigma-Aldrich (St. Louis, MO).

### Histological analysis

At the end of experiments, the trachea catheter was connected to a Harvard rodent ventilator (Model 683; Harvard Apparatus, South Natick, MA, USA), the thorax was opened, and the lungs were flushed with saline (0.9% sodium chloride) to remove the blood from the pulmonary vessels. Animals were euthanized by the anesthetic overdose (pentobarbital 200 mg/kg), and the heart and lungs were dissected and weighed. The right ventricle (RV) free wall was separated from the left ventricle (LV), and the septum (S) and the Fulton index (RV/ LV + S) was calculated. The total wet lung weight was measured and normalized by the body weight of the animal. Lower right lobe was removed, weighed, and stored for 18 h at 65 C^0^. The dry weight was measured and wet-to-dry weight ratio calculated. One lung was immersed in 10% buffered formalin for at least 72 h before being embedded, whereas the other was stored at − 80 °C and used for biochemical studies. Four-micrometer serial tissue sections from paraffin-embedded lungs were dewaxed and stained with hematoxylin and eosin (H&E), Verhoeff’s-Van Gieson (VVG) and Pentachrome staining for histological and morphometric assessment. Perivascular cuffs area was blindly calculated by subtraction of Media vessel area from the Cuff area. The data from Day 12 and 24 were based on vessels size 50–100 and 100–300 um. The Aperio ScanScope XT system (Aperio Inc., Vista, CA, USA) was used to scan entire glass slides at 20× magnification, and Aperio ImageScope and ImageJ software were used to analyze digital slides.

### Plasma protein nitration in rat and human samples

Dot-blot analysis was performed to measure the total level of plasma protein nitration as previously described [21] with modifications. Briefly, 1 μl of diluted plasma samples were spotted onto a nitrocellulose membrane (Bio-Rad). The membrane was then allowed to dry overnight at room temperature, blocked with 1% of BSA in TBS containing 0.1% Tween 20 for 1 h, and incubated with antibodies specific for either 3-nitrotyrosine (3-NT; 1:2000; Calbiochem) for 2 h at room temperature. After being washed three times, the membranes were incubated with secondary antibody, washed, visualized using the chemiluminescent procedure, recorded using a Li-Cor Odyssey FC Imager and analyzed using IS Image Studio 5.0 software. For measuring plasma protein nitration in rat samples, the obtained signal was normalized per total plasma protein by using the 1 μg of plasma protein/μl of spotted sample in all rat samples. For patient samples, the obtained protein nitration signal was normalized to the total plasma protein amount measured by Ponceau S stain and quantified by Image J software.

### Human subject’s blood collection

All subjects provided written consent to participate in this study with the approval of the University of Arizona (UA) institutional human subjects review board. The study cohort consisted of patients with a right heart catheterization (RHC)-confirmed diagnosis of Group I PAH and were prospectively recruited from the UA PAH clinic from 2013 to 2015. Blood from 12 male patients and 29 female patients was drawn during outpatient clinic visits or during an electively scheduled RHC (from an antecubital vein). Care was taken to standardize blood sample collection, preparation and storage at –80 °C as described [36].

### Glycolytic intermediates analysis

Data were acquired using the West Coast Metabolomics Center at UC Davis. Briefly, Restek corporation Rtx-5Sil MS column was used with helium mobile phase at temperature interval (50–330 °C) and flow-rate: 1 mL min^− 1^. Injection volume was 0.5 μL at 50 °C ramped to 250 °C by 12 °C s^− 1^. Oven temperature started at 50 °C for 1 min, then ramped at 20 °C min^− 1^to 330 °C, and held constant for 5 min. Mass spectrometry analysis was done on a Leco Pegasus IV mass spectrometer with unit mass resolution at 17 spectra s^− 1^ from 80 to 500 Da at − 70 eV ionization energy and 1800 V detector voltage with a 230 °C transfer line and a 250 °C ion source. Raw data files were preprocessed directly after data acquisition and stored as ChromaTOF-specific *.peg files, as generic *.txt result files and additionally as generic ANDI MS *.cdf files. ChromaTOF vs. 2.32 was used for data preprocessing without smoothing, 3 s peak width, baseline subtraction just above the noise level, and automatic mass spectral deconvolution and peak detection at signal/noise levels of 5:1 throughout the chromatogram. Apex masses were reported for use in the BinBase algorithm. Result *.txt files were exported to a data server with absolute spectra intensities and further processed by a filtering algorithm implemented in the metabolomics BinBase database.

The data were represented as peak heights for the quantification ion (mz value) at the specific retention index. Final data were plotted in the GraphPad Prism software.

### Statistical analysis

Results are presented as Mean ± SEM. Statistical analyses were performed using GraphPad Prism V. 4.01 software. Group comparisons were carried out using one-factor nested (hierarchical) analysis of variance (1F-ANOVA). If ANOVA indicated a significant difference among the means, specific comparisons were made with Newman-Keulus multiple comparison tests. Alternatively, the unpaired *t*-test between two specific animal or cell treatments groups was used. The statistical analysis used in each figure and the number of replications are indicated in the figure legend. A value of *P* < 0.05 was considered significant. An analysis of the correlation between patient plasma 3NT levels and cardiac output or Pulmonary Vascular Resistance (PVR) and animal lung glucose levels and RVSP were performed using two-tail test of the linear regression model. The Pearson coefficient and *P* values calculated by GraphPad Prism V. 4.01 software are presented on the corresponding figures.

## Results

### Repetitive inhibition of mitochondrial respiration induces a sustained increase in right ventricle pressure

**Fig. 1:**
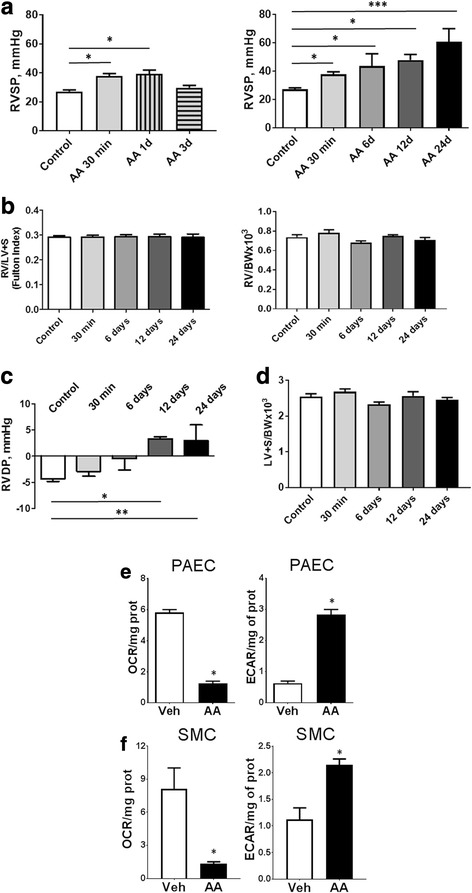
Acute and chronic right ventricle hemodynamics and remodeling in Antimycin A-treated rats. RV systolic (**a**) pressure in untreated Controls and acutely AA-treated rats 30 min, 1 and 3 days after AA (0.35 mg/kg) injection show the acute and reversible effect of one dose of AA on pulmonary pressure (left panel). Right panel represents RV systolic pressure in chronically treated rats. 6, 12 and 24 days after AA injection show a progressive chronic pulmonary vasoconstriction and RV dysfunction. There was no evidence of RV hypertrophy during the first 24 days of AA treatment (**b**). End diastolic RV pressure indicates significant elevation at day 12 and 24 (**c**). There is no indication of LV hypertrophy (**d**). AA (10 μM) treatment of PAEC (**e**) or SMC (**f**) results in a 6-fold decrease in OCR. Decrease in OCR was accompanied with an increase in glycolysis as indicated by ECAR. Data are mean ± SEM, *N* = 4–13 per group. *P < 0.05 vs. Control/Vehicle in 1-way ANOVA or t-test for two groups

The injection of AA-induced a mild but significant increase in RVSP even 30 min after the first injection (Fig. 1a left panel). However, this acute vasoconstrictive response was lost on day 3 after the first injection (Fig. 1a left panel). The second dose, given on day 3, promoted a prolonged increase in RVSP even 3 days after the second injection (or day 6 since initiation of treatment). Finally, the third dose injected on day 6 ensured the RVSP to stay significantly increased and continue to elevate during the next 18 days after the last injection (or on days 12 and 24 since initiation of study) (Fig. 1a right panel). While we suggest that the acute AA response may be due to an ability of superoxide to scavenge NO or induce the calcium signaling in the smooth muscle cells [37], we proposed that in the more advanced stages, and especially on day 12 and day 24, other mechanisms are responsible for chronic vasoconstriction. Some of these mechanisms were uncovered in this study.

Surprisingly, while chronic AA treatment induced an increase in RV pressure, the Fulton index and RV normalized on body weight in all experimental groups remained unchanged (Fig. 1b). At the same time, although the AA did not induce an RV hypertrophy, the RV diastolic function was found to be affected. Thus, the RV end Diastolic Pressure (RVDP) showed a progressive increase and became significantly higher by day 12 and day 24 compared to controls (Fig. 1c). Importantly, Left Ventricle (LV) was not affected throughout the course of the study (Fig. 1d).

By treating rat Pulmonary Artery Endothelial Cells (PAEC) and Pulmonary Artery Smooth Muscle Cells (PASMC) with the same dose of AA used to initiate pulmonary vasoconstriction in vivo*,* we have confirmed that this dose AA is capable to efficiently inhibit mitochondrial respiration in pulmonary vascular cells. Our data from Seahorse XF analyzer indicated a 6-fold decrease in Oxygen Consumption Rate (OCR) in both PAEC and PASMC (Fig. 1e, f). Opposing to OCR, the ExtraCellular Acidification Rate (ECAR) that reflects lactate production due to glycolysis was increased by 6-fold in PAECs and 2-fold in PASMC. These data indicate that PAEC are more readily switch to glycolysis, while PASMC with inhibited mitochondrial respiration are less compensated in the energy demand compared to endothelial cells.

### AA treatment produces chronic vasoconstriction and perivascular edema

**Fig. 2:**
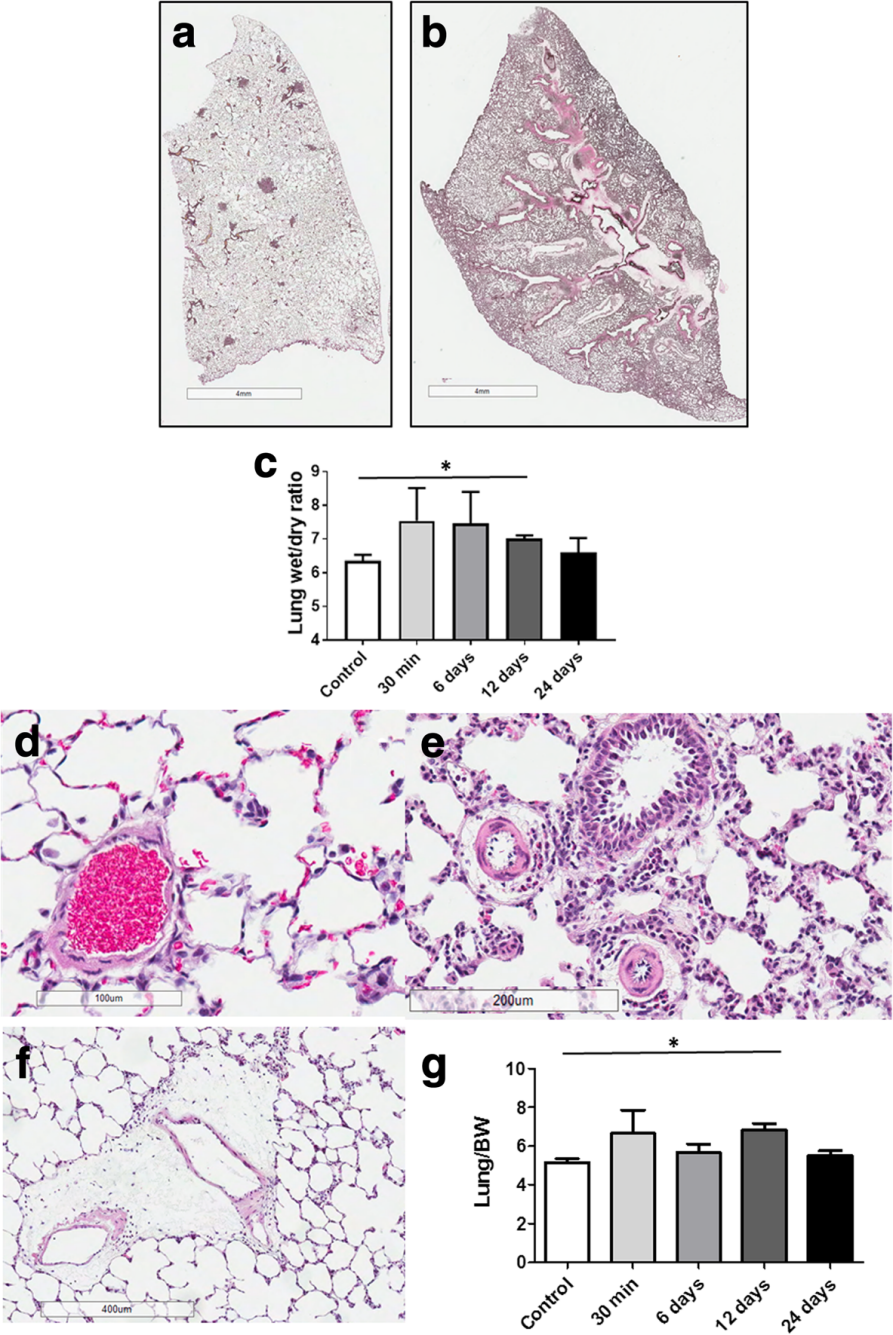
Antimycin A induced pulmonary injury on Day 12. Photomicrographs of whole lob section of lung tissue from control (**a**) and Antimycin A 12 days challenged lungs (**b**). Lung wet to dry ratio indicates significantly increased edema at day 12 (**c**). Normal perivascular structure in control animal (**d**) and marked (**e**) and severe (**f**) pulmonary perivascular edema in AA-treated rats at Day 12. Although the total wet lung weight normalized by body weight shows an increase right after AA injection, this acute lung edema was not significant (**g**). However, by day 12 the difference in the lung weight as a measure of pulmonary edema became significant compared to Control group (**g**) H&E staining. Photomicrographs are representative of 5 lung preparations per experimental group. For lung edema evaluation the data are mean ± SEM, *N* = 4–6 per group. *P < 0.05 vs. Control using unpaired *t*-test

The repetitive administration of AA increased the overall size of the lung seen upon examination of the whole lobe section in AA-treated rats compared to Control (Fig. 2a, b), as well as promoted lung vascularization (dark pink staining) and induced severe perivascular edema (light pink areas). Lung wet to dry ratio was increased acutely at 30 min and chronically at day 6 and 12, however, significance was reached at day 12 only and was resolved by day 24 (Fig. 2c). Normal perivascular structure in control animal (Fig. 2d) transformed to marked (Fig. 2e) and severe (Fig. 2f) pulmonary perivascular edema in AA-treated rats at day 12. The wet lung weight normalized to the body weight showed an early, but not significant, increase after acute AA injection and the second wave of significant lung weight increase on day 12 (Fig. 2g).

**Fig. 3:**
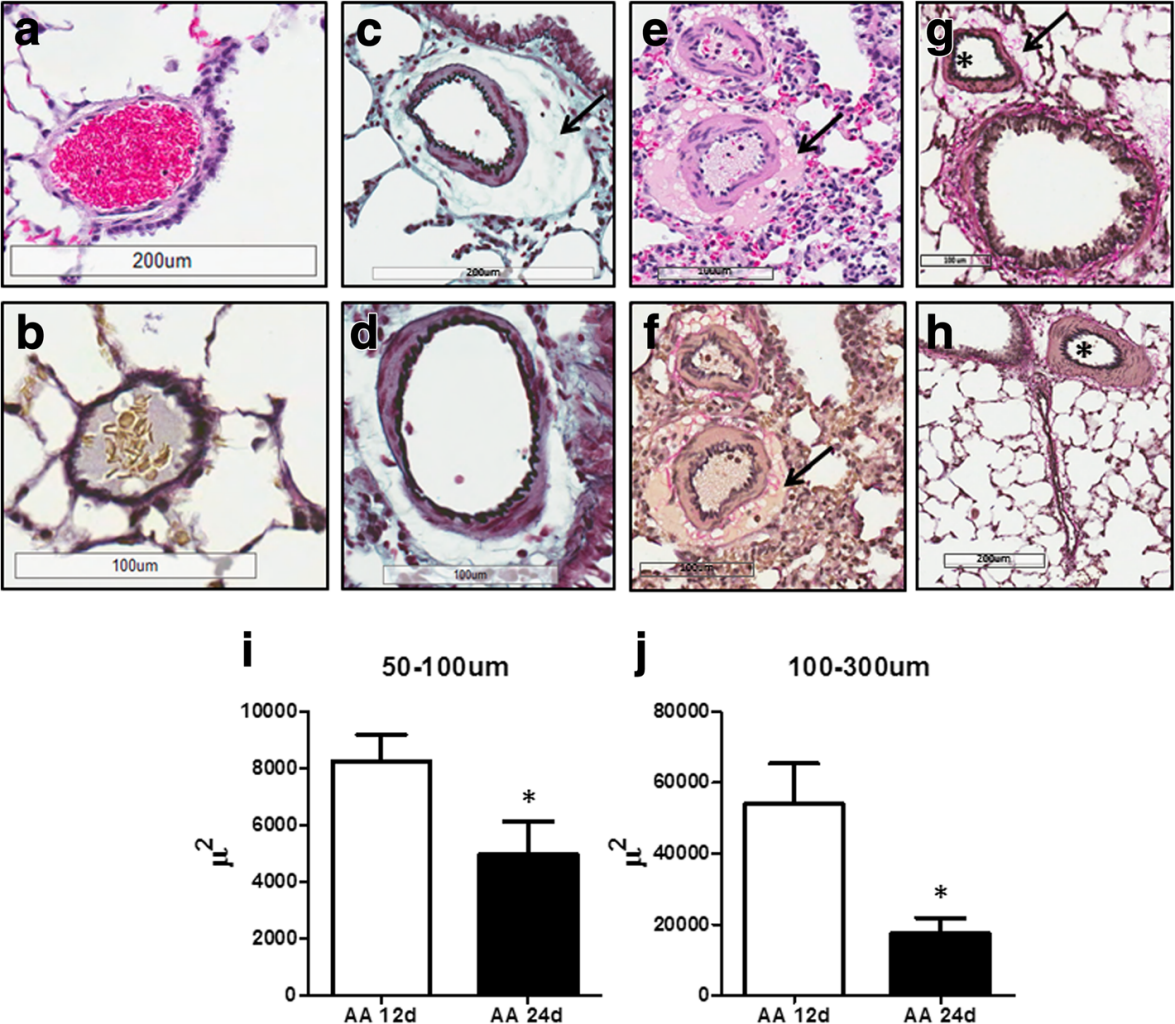
Small-size pulmonary arteries from Control (**a** and **b**) and Antimycin Day 12 (**c** and **d**) and Antimycin Day-24 (**e**, **f**, **g**, **h**) rats. Asterisks indicate vasoconstriction and arrows denote the presence of perivascular cuff indicative of endothelial barrier dysfunction. H&E staining: **a** and **e**; Verhoeff-Van Gieson (VVG) staining: **b**, **f**-**j**; Pentachrome staining **c** and **d**. Photomicrographs are representative of 5 lung preparations per experimental group. Scale bar indicate 200 μm for **a**, **b**, and **h** and 100 μm for **c**, **d**, **e**, **f**, and **g**. Quantification of perivascular edema area showed a significant decrease in both small (**i**) and large (**j**) pulmonary arteries in day 24 group compared with day 12. Data are mean values ± SEM, *N* = 9–17 per group. *P < 0.05 vs. AA 12 days using unpaired *t*-test

Moreover, the perivascular edema of small pulmonary arteries (negative in control animals Fig. 3a, b) appeared to be a hallmark of the late effect of AA, especially seen on day 12 (Fig. 3c, d, e, f). These results are in great accordance with the changes in the lung weight (Fig. 2f) and lung wet/dry ratio (Fig. 2c).

However, by day 24 perivascular fibrosis, an occasional vascular remodeling and pronounced vasoconstriction continued to progress, as evidenced by Verhoeff-Van Gieson (VVG) staining (Fig. 3g, h). Our quantification of small (Fig. 3i) and large (Fig. 3j) pulmonary arteries perivascular cuffs area at day 12 and 24 exhibited that edema was less evident at day 24.

### The levels of plasma protein nitration correlate with PAH progression in female AA-treated rats and female PAH patients

**Fig. 4:**
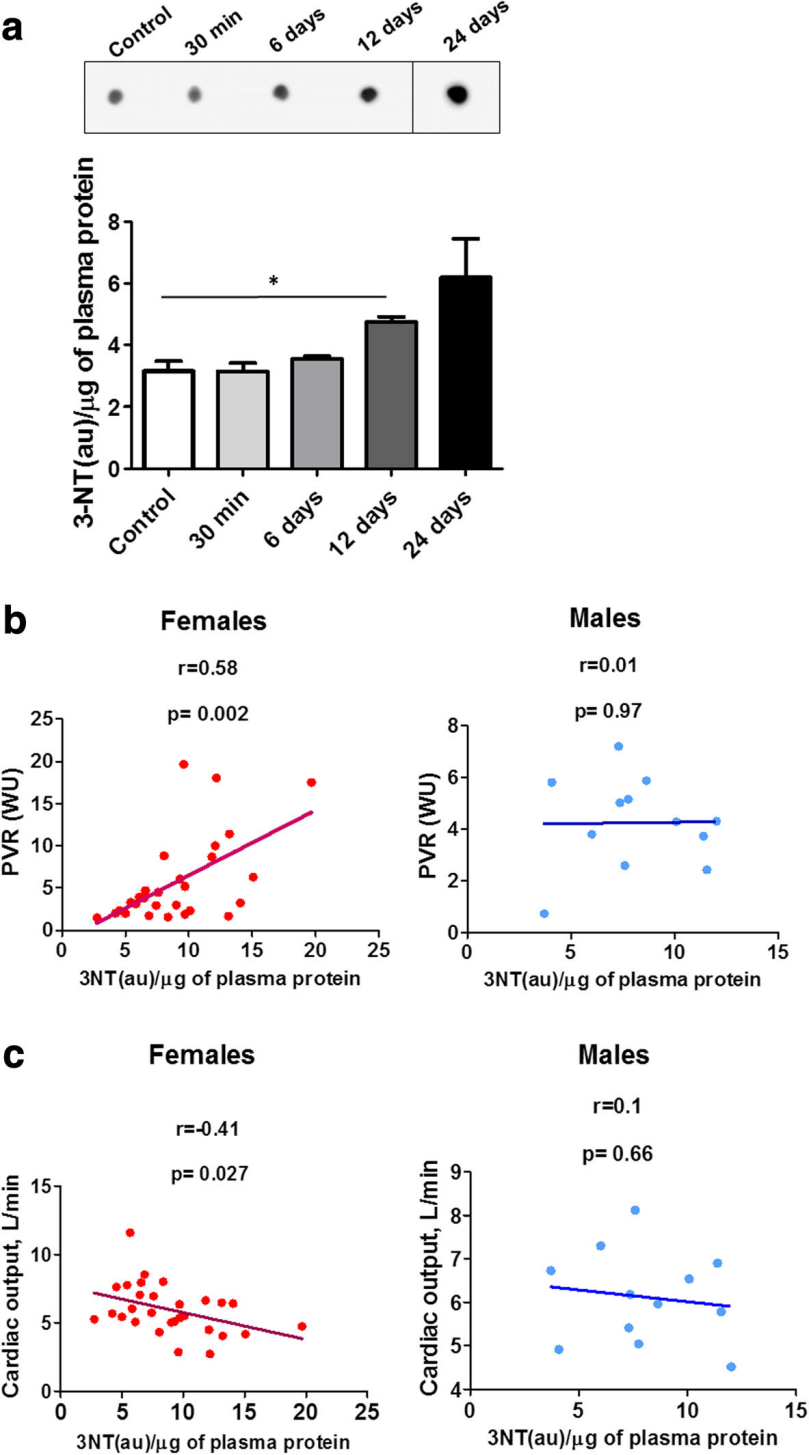
Total protein nitration in plasma samples of AA-treated rats and from PAH patients. Densitometric analysis indicates that AA injections induced an accumulation of plasma nitrated protein, which become significant by day 12 of study (**a**). Representative dot for day 24 was taken from different part of the same membrane. The levels of the plasma protein nitration in females but not male patients with PAH positively correlate with pulmonary vascular resistance (PVR, WU, **b** and negatively – with cardiac output (L/min, **c**), suggesting the nitrosative stress could be involved in PAH progression in females. **a** - data are mean ± SEM, *N* = 3–5 per group. *P < 0.05 vs. Control using unpaired *t*-test; **b**, **c** - *N* = 12–29 per group, Person coefficient and *P*-value were calculated by two-tail correlation test

The progression of AA-induced pulmonary vasoconstriction was associated with an accumulation of nitrated proteins in the plasma (Fig. 4a). The protein nitration was not evidenced after an acute AA injection and produced only a very mild effect on day 6, however, at day 12 the increase became significant. Although the statistical significance was not reached in day 24 group due to the high variability among the animals, there was an overall trend of further elevation, suggesting that there may be an association between the levels of protein nitration and PAH progression. Indeed, when the protein nitration was evaluated in PAH patients, it revealed a strong positive correlation with pulmonary vascular resistance (PVR), a key marker of PAH severity, in only female patients (Fig. 4b). There was also a significant negative correlation between nitration of the plasma proteins and a decline in cardiac output (CO) as a measure of RV dysfunction, again, only in female but not male patient groups (Fig. 4c).

### Inhibition of mitochondrial respiration produces a severe metabolic shift in lungs

**Fig. 5:**
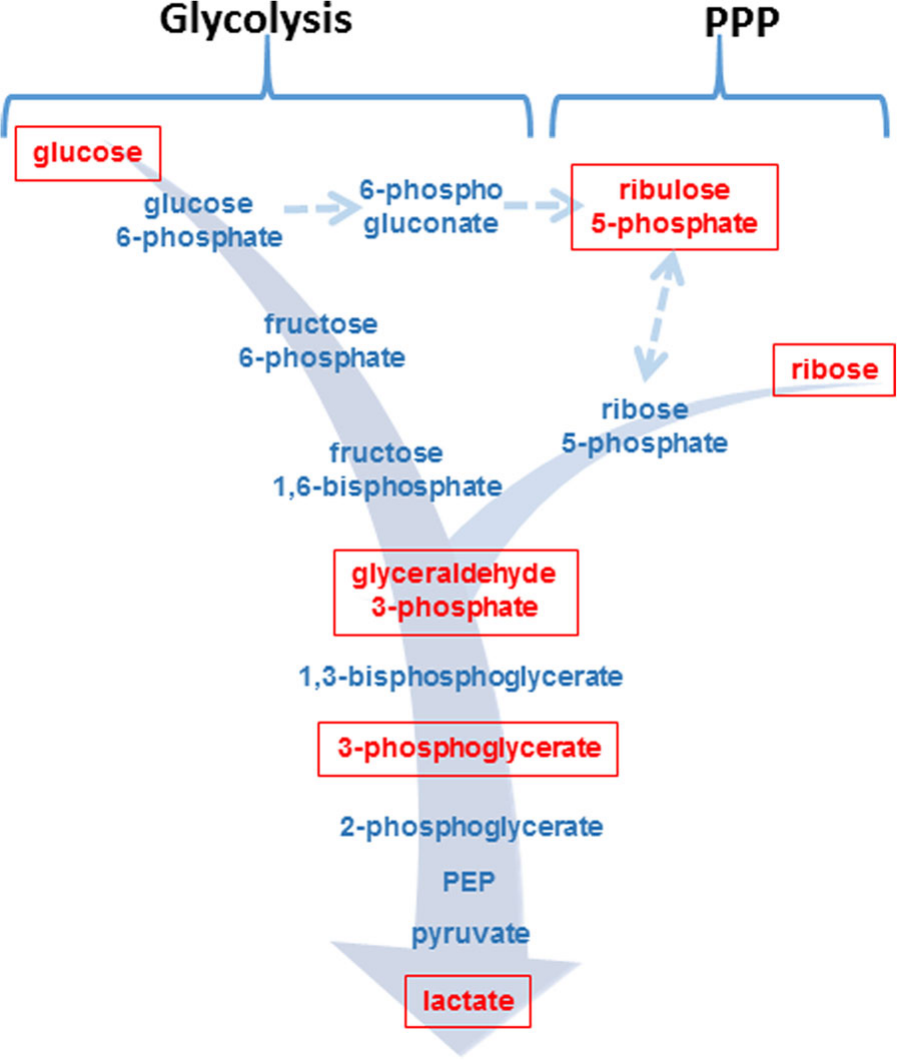
Analysis of glycolytic switch in Antimycin A-treated rats. To analyze the glycolytic flux in AA-treated rats, we monitored the stable intermediates in the glycolysis and pentose phosphate pathways indicated as red

**Fig. 6:**
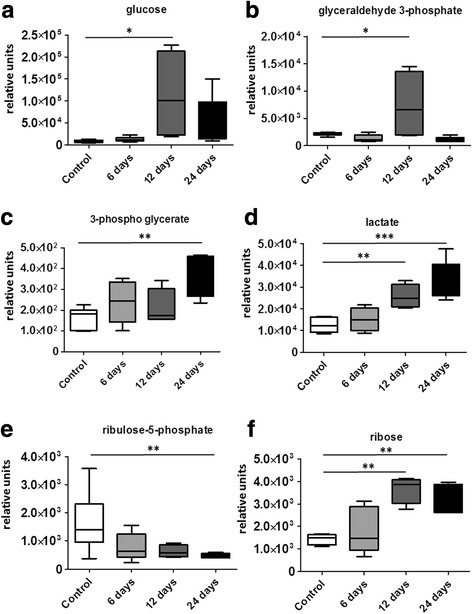
Chronic electron transport chain inhibition induced a glycolytic shift in lungs. Our data indicate accumulation of glucose (**a**) and glyceraldehyde 3-phosphate (**b**) at day 12 of study. In contrast, 3-phosphoglycerate (**c**) and final product of glycolysis – lactate (**d**) – possess a progressive increase that becomes significant by day 24 for 3-phosphoglycerate and both day 12 and 24 for lactate. However, an upstream pentose phosphate intermediate – ribulose-5-phosphate (**e**) has gradually decreased with a disease progression with a significant reduction by day 24. Finally, the content of ribose (**f**) in the lungs was also strongly elevated at day 12 and 24, which may represent the ribose to glyceraldehyde-3-phosphate shuttle that contributes to upregulation of pulmonary glycolysis. Data are mean ± SEM, N = 4–7 per group. *P < 0.05 vs. Control in 1-way ANOVA

It has been previously reported that the lungs of both PAH patients and PH animals show a metabolic reprogramming and transition to glycolysis [30–33]. To evaluate whether chronic AA injection could mediate the same metabolic shift, we analyzed a few primary metabolites in the lungs of rats on day 6, 12 and 24. The analyzed metabolites were specifically chosen to identify changes in the glucose/ribose pathway (Fig. 5) and evaluate the level of glycolytic changes in the lungs after chronic ETC inhibition. We found that all analyzed metabolites possessed a strong increase in the lungs of AA-treated rats. However, the extent and the specific profile of metabolic changes varied at different time points. Thus, there was an increase over 13.9 times in the level of glucose and an increase over 3.4 times in glyceraldehyde 3-phosphate on day 12 compared to control animals (Fig. 6a, b). At the same time the downstream metabolite 3-phosphoglycerate was significantly accumulated only on day 24 (2.2 times increase vs. Controls) and lactate, the final product of glycolysis, was elevated on both 12 and 24 days (2.1 and 2.6 times increase respectively, Fig. 6c, d). Importantly, the pulmonary levels of ribulose-5-phosphate were found to be only decreased (Fig. 6e), suggesting that glucose was primarily used in the glycolytic pathway, while the pentose phosphate pathway (PPP) became inhibited (Fig. 5). We found that ribose accumulation (Fig. 6f) may contribute to the glycolysis flux by the shunt into glyceraldehyde 3-phosphate.

### Pulmonary glucose strongly correlates with the level of pulmonary vasoconstriction

**Fig. 7:**
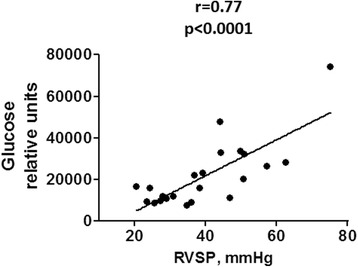
Correlation between pulmonary glucose levels and right ventricle systolic pressure. The strong correlation between the RVSP and glucose levels in all AA groups analyzed together suggest the contribution of metabolic reprogramming in lungs upon AA treatment in chronic pulmonary vasoconstriction. *N* = 25; Person coefficient and *P*-value were calculated by two-tail correlation test

To evaluate whether the discovered strong increase in lung glucose levels was associated with the disease progression, we analyzed the correlation between the lung glucose levels and RVSP in all animal groups together. We found a very robust (*r* = 0.77) and significant (*p* < 0.0001) correlation between these two parameters in our animal model (Fig. 7). These data in a strong accordance with the previously published research proposed that PAH progression is mediated by a shift in the cellular metabolism from glucose oxidation toward glycolysis.

## Discussion

There is a vast body of literature from animal models to patients with PAH confirming that different types of mitochondrial dysfunction are strongly associated with PAH pathobiology [1, 2, 30, 31]. However, whether these alterations are directly triggered PAH or subsequent manifestations of the disease and contribute to PAH progression remains unclear. The current study demonstrates, for the first time, that the chronic inhibition of ETC is sufficient to initiate sustained pulmonary vasoconstriction, an increased vascular edema, and a glycolytic metabolic shift in healthy female rats. These observations strongly support the notion that impaired mitochondrial respiration can trigger PAH development.

Interestingly, when the effect of Crotalaria spectabilis plant seeds given orally was discovered to produce experimental PH in the 1960s, the early effects were described as acute pulmonary edema, infiltration of mononuclear cells and eosinophils, and pulmonary arteritis with edema of the pulmonary artery wall [38, 39]. Pulmonary edema at early stages of PAH (14 days after MCT i.p. injections) was also observed in another recent study, occurring at different anatomic levels (vascular edema, perivascular, bronchial, peribronchial, and alveolar septae) [40]. By day 28 after MCT injection, this edema was found to turn into vascular remodeling, suggesting that MCT induced damage of pulmonary arteries and subsequent edema may contribute to the initiation of uncontrolled vascular repair and remodeling at late stages. In this study, we found that chronic ETC inhibition also induces perivascular edema, which peaks at day 12 and mimics the histological changes seen in MCT and Sugen/hypoxia (our unpublished observation) models. We suggest that perivascular edema could contribute to vascular stiffness, impair the oxygen transport to the vascular wall and potentiate the activation of inflammatory cells. All these events (vascular stiffness, hypoxia, and inflammatory component) are known to be tightly associated with the increase in the pulmonary arterial pressure and contribute to the pathogenesis of PAH. Besides, this early edema may promote a late onset of vascular proliferation in PAH. Indeed, a subset of pulmonary arteries at days 12 and 24 has displayed the signs of remodeling. These observations support the idea that AA treatment is capable of inducing progressive pulmonary vasoconstriction even 18 days after the last AA application. The development of early lung edema may reflect either a direct deleterious effect of AA on pulmonary endothelial cells or initiated by an enhanced mitochondrial ROS production, which is known to induce p38 MAPK mediated endothelial barrier disruption [41]. Following the histological developments in these rodent models for a longer period of time is likely required to confirm this observation.

In this study, the chronic AA treatment did not result in the development of RV remodeling. While the occasional severe PAH without a detectable RV hypertrophy has been previously described for humans [42] and reported in a mouse model with PAH phenotype [43], the absence of RV hypertrophy did not allow us to consider the AA-induced condition as a developed pulmonary hypertension. Instead, we classify the AA-induced changes as an early stage of the disease mainly characterized by chronic pulmonary vasoconstriction. Nevertheless, the absence of RV hypertrophy may be attributed to the inhibitory effect of ETC dysfunction on myocardial contractility. In particular, the reduced levels of ATP production upon ETC inhibition, for example, can diminish RV reserve without an ability to respond to elevated afterload with hypertrophic remodeling. At the same time, the progressively increased RV afterload in combination with reduced RV contractility may be the primary reason for an elevated end-diastolic volume, potentially contributing to an elevated RVDP (Fig. 1c).

The high levels of NO bioavailability in females were expected to induce acute nitrosative stress via an increase in mitochondrial superoxide production induced by ETC inhibition. However, our protein nitration data were not revealing elevations at 30 min after AA injection. In contrast, there was a progressive accumulation of nitrated plasma proteins that correlated with a gradual disease progression. These data suggest that basal NO production may be insufficient to induce an increase in plasma protein nitration, although it may still produce higher levels of protein nitration in specific cell compartments. However, the progressive chronic vasoconstriction induced by AA treatment may be responsible for the further stimulation of NO production, which, on the background of superoxide leak, could initiate sustained nitrosative stress and may contribute to PAH progression. This notion was supported by a strong positive correlation between the total protein nitration in the plasma samples of female PAH patients and PVR, and a negative correlation with CO, which both are important markers of PAH severity (Fig. 4). We conclude that the increased level of NO bioavailability associated with female gender promotes accumulation of nitrated proteins specifically in female patients. Nevertheless, whether the observed nitrosative stress contributes to PAH progression or just could serve as a gender-specific marker of PAH progression remains to be elucidated.

PAH development and progression are characterized by changes in small circulation hemodynamics, lung and right heart morphology, altered signaling, transcriptomics, proteomics, and metabolism. We have recently reported that before an alteration in macro-parameters such as morphology and hemodynamics, we have observed metabolic changes, which predisposed organs to the pathological transition [34]. The glycolytic switch of metabolism in pulmonary vasculature is a well-accepted and characterized phenomenon in PAH. It was shown with a PET scanning of PAH patients that increased glycolysis in lungs and RV, similar to glycolytic changes seen in the tumors of cancer patients, correlates with the presence and severity of PAH [44, 45]. However, there is still debate whether the glycolytic switch is secondary to electron transport chain dysfunction or is upregulated due to pathologic normoxic HIF1a signaling activation. Our data indicate that ETC inhibition can turn on the glycolytic shift in the lungs. Indeed, AA-induced mitochondria dysfunction led to a robust upregulation of glucose uptake in the lung and resulted in an accumulation of several glycolytic pathway intermediates. Besides, we found that although based on ribulose-5-phosphate data, the PPP pathway was reduced in our animals; the ribose level was upregulated and can be fed into the glycolytic pathway via ribose conversion by ribokinase. It is important to note that glycolysis is elevated 12 days after induction of mitochondrial dysfunction but reduced at the late stage (24 days). Although primary glycolytic pathway is reduced at day 24, the lactate levels are still elevated. This can be explained by an activation or overexpression of ribokinase that can convert ribose via increased 3PG into production of lactate. Thus, AA-induced mitochondrial dysfunction can lead to the similar metabolic changes observed in other pre-clinical models and PAH patients.

Aerobic glycolysis is not a very efficient way for ATP generation when compared to the amount produced by mitochondrial respiration. However, the rate of glucose metabolism via glycolysis is higher than the complete oxidation of glucose in the mitochondrial respiratory chain [46]. Moreover, glycolysis induces a carbon flux toward the production of intermediates that are needed for biosynthesis of lipids, amino acids, porphyrins, and nucleotides leading to uncontrolled proliferation [47]. An upregulation of glycolysis is known to induce anabolic reprogramming of cells, leading to a higher rate of cell proliferation. Thus, the upregulation of glycolysis in response to mitochondrial dysfunction may become a key factor in vascular remodeling that would maintain the increased pulmonary resistance and pulmonary pressure [48]. It is important to emphasize that glucose uptake by lungs of AA-treated rats was found to strongly correlate with a pulmonary pressure, confirming the importance of glycolysis in PAH progression.

## Conclusion

In conclusion, we believe that this study provides a novel and important insight into the pathogenesis of PAH. The results of our study suggest that the initial mitochondrial dysfunction is involved in the early changes associated with PAH, such as pulmonary vasoconstriction, vascular edema, and metabolic shift. Although these early pathological events have been previously described for other models of PAH, they have never been observed in response to direct mitochondrial inhibition. While further research is required to fully understand the role of mitochondria in the complex mechanisms of PAH initiation and progression, our study provides the novel evidence of the causal role of mitochondrial dysfunction in the initiation of pathological events associated with PAH.

## Abbreviations

3NT: 3-Nitrotyrosine
AA: Antimycin A
ANOVA: Analysis of variance
ATP: Adenosine triphosphate
BSA: Bovine serum albumin
Cav1: Caveolin 1
DNA: Deoxyribonucleic acid
ECAR: Extracellular acidification rate
eNOS: endothelial nitric oxide synthase
ETC: Electron transport chain
H&E: Hematoxylin and eosin
LV: Left ventricle
MCT: Monocrotaline
MnSOD: Manganese Superoxide dismutase
mROS: mitochondrial reactive oxygen species
NFU1: NifU-Like C-Terminal Domain Containing protein
NO: Nitric oxide
OCR: Oxygen consumption rate
PAH: Pulmonary arterial hypertension
PE: Polyethylene
PET: Positron Emission Tomography
PPH: Primary pulmonary hypertension
PPP: Pentose phosphate pathway
PVR: Pulmonary Vascular Resistance
RHC: Right heart catheterization
ROS: Reactive oxygen species
RV: Right ventricle
RVDP: Right ventricular diastolic pressure
RVSP: Right ventricular systolic pressure
S: septum
TBS: Tris-Buffer-Saline
VVG: Verhoeff’s-Van Gieson
